# Experiences and Expectations of Patients Hospitalized for COVID-19: A Qualitative Study in Poland

**DOI:** 10.3390/ijerph19052992

**Published:** 2022-03-04

**Authors:** Ludmila Marcinowicz, Ewa Fejfer-Wirbal, Ewa Taranta, Slawomir Chlabicz, Slawomir J. Terlikowski

**Affiliations:** 1 Department of Obstetrics, Gynecology and Maternity Care, Medical University of Bialystok, 15295 Bialystok, Poland; sterlikowski@gmail.com; 2 Department of Health Protection, State Vocational University in Suwalki, Noniewicza 10, 16400 Suwalki, Poland; e.wirbal@pwsz.suwalki.pl; 3 Non-Public Health Care Center “Fidos” Family Clinic, 15182 Bialystok, Poland; taranta59@wp.pl; 4 Department of Family Medicine, Medical University of Bialystok, 15054 Bialystok, Poland; schlabicz@poczta.onet.pl

**Keywords:** COVID-19, patients, qualitative research, Poland

## Abstract

The COVID-19 pandemic has caused many new problems and challenges for medical personnel, patients and their families. The present study aimed to learn the difficulties and expectations of patients hospitalized for COVID-19. A descriptive qualitative research approach was adopted, and the study was carried out using semi-structured telephone interviews with 20 patients according to Consolidated Criteria for Reporting Qualitative Research (COREQ) guidelines. Two main themes were extracted from the experiences related by patients hospitalized for COVID-19: difficulties resulting from their poor health condition and difficulties resulting from hospital conditions and safety protocols. The patients’ expectations referred to professionalism and family members’ support. From the patient’s perspective, a sense of humor in the care provider is important, apart from professionalism and the effectiveness of treatment, because humor helps the patient endure difficult situations. The findings indicate that patients hospitalized for COVID-19 experienced both positive and negative emotions. Their negative experiences concerned organizational barriers and medical personnel shortages, especially of clinical nurses. Patients’ expectations are realistic and appropriate to the situation they are in. Learning the difficulties and expectations of patients hospitalized for COVID-19 may help care providers cope with this disease more effectively and ensure better care for patients, including nursing and psychological services.

## 1. Introduction

The COVID-19 pandemic has caused many new problems and challenges for medical personnel as well as for patients and their families. Previous research results show that the psychological experiences of patients hospitalized for COVID-19 included fear, denial and stigma, and the main sources of stress were the viral character of the disease, quarantine, and concerns about the health of their family members [1]. Patients with COVID-19 experience negative emotions after discharge from hospital, a need for social and psychological support, and many fears connected with their return to functioning in society [2]. Hospitalized patients also experience loneliness, which calls for compassion and empathy from medical personnel [3].

The first case of SARS-CoV-2 infection in Poland was confirmed on 4 March 2020. According to data compiled by the World Health Organization (WHO), from the beginning of the pandemic until the end of August 2021 there were 2,888,670 cases of COVID-19 and 75,345 deaths in Poland. Globally, 216,867,420 cases and 4,507,837 deaths were confirmed in the same period [4].

Owing to the epidemiological situation, the Polish government introduced a number of bans, orders, and restrictions. There were also changes in the functioning of the health care system. For example, telephone consultations with doctors were introduced into primary health care, and in-person contact with a doctor was possible only after the telephone consultation and upon meeting certain conditions. In hospitals, the number of beds for patients with COVID-19 was increased, and other units were converted to COVID wards. In each province, some specialist hospitals were transformed into COVID referral hospitals. As the epidemic was intensifying, some hospitals were also designated as COVID referral hospitals. Some provisional, reserve hospitals were also set up in public buildings [5]. These changes caused an increased demand for medical staff as well as an excessive burden on them resulting from greater responsibilities and difficulties associated with working under high-risk conditions.

One of the important patient challenges during the COVID-19 pandemic was the introduction of restrictive rules for visiting hospitalized patients and the prohibition of access to hospital wards for families and care givers. This adversely impacted the much-needed comfort and support that hospitalized patients typically receive from family and friends [6]. Restrictions also applied to spiritual care practitioners, who complained that they did not have time to talk to patients, had problems administering the sacraments, and often had to limit their ministry [7].

Understanding patients’ perceptions of hospital care as the COVID-19 pandemic continues will play a significant role in decision making and restructuring of hospital services to improve patient care. Listening to patients and collecting and analyzing data on patient experiences are essential to maintaining and improving the quality of medical and spiritual care. Medical professionals need new skills to ensure safe care during a pandemic [8].

Qualitative studies concerning the experiences of patients hospitalized for COVID-19 are scarce and cannot be generalized. Due to the limited number of studies on the experiences of hospitalized patients, we sought to fill this gap by studying the perspectives of Polish patients. The aim of the study was to learn the difficulties and expectations of patients hospitalized for COVID-19.

## 2. Materials and Methods

A qualitative study was carried out using the interview technique [9] and applying Consolidated Criteria for Reporting Qualitative Research (COREQ) guidelines [10].

### 2.1. Study Design and Data Collection

We used the descriptive qualitative research approach as the method of choice to succinctly describe in everyday terms the experiences related by patients [11]. Semi-structured interviews were carried out according to an interview guide. The interview guide included the following questions: What was your hospital stay like? What was the most difficult aspect for you? How did the hospitalization affect you? How did your hospitalization affect your family?

The interviews were carried out in the first half of 2021.

### 2.2. Participants and Study Settings

We used convenience and purposeful sampling to recruit patients with a range of demographics and hospitalization periods. The participants were recruited from three primary health care centers at which they were patients. They were invited to participate in the study over the phone. The interviews continued until data began to repeat and we achieved data saturation. We finished collecting the data when there were no new threads in the patients’ statements.

The study participants were hospitalized in three public hospitals in the north-eastern region of Poland with different levels of referral. Due to the lack of places in infectious disease wards, some stayed in wards temporarily adapted for the treatment of COVID-19.

### 2.3. Interview Procedure

The interviewer (the first author) phoned each patient, presented the aim of the study, and asked them whether they would be willing to participate in the study. If the patient agreed, then a phone interview at a time convenient for the patient was arranged. At the agreed time, the interviewer phoned the patient again and conducted the interview. Patients expressed their oral consent to participate in the study; it was recorded at the beginning of the interview. No patient refused to participate. All interviews were audio recorded and transcribed verbatim. The interviewer has extensive experience in carrying out qualitative research.

### 2.4. Data Analysis

All interviews were analyzed by two authors (LM and EF-W) using thematic analysis. Transcripts were coded manually line by line using the initial framework. Data within each category were then analyzed by the same two researchers to create sub-categories. To ensure rigor, the ongoing analysis was discussed within the study team.

To ensure the accuracy, validity and reliability of the data, we applied member checking [12]. That is, we invited four participants to provide feedback on our preliminary version of the results. A report from the study was sent to those individuals by e-mail to read and comment on. All four participants agreed with our interpretation of the results, and one person added this comment: “The only reflection after reading this is that age is key in how you are affected by this disease. The older you are, the greater the stress” (male, age 37, seven days in hospital).

### 2.5. Ethical Issues

The consent of the Bioethics Committee of the Medical University of Bialystok was obtained for the study (no. APK.002.414.2020).

### 2.6. Characteristics of the Study Participants

Study participants were 20 patients hospitalized for COVID-19, 7 women and 13 men. The participants’ mean age was 57 (the youngest person was 32 years old, and the oldest, 75). Ten participants had secondary education, eight higher education, and two vocational education. The shortest time of hospitalization was 5 days, and the longest, 49 days (Table 1).

## 3. Results

### 3.1. Most Difficult Situations during Hospitalization

Analyzing patients’ responses concerning the most difficult situations during their hospitalization, we determined two main categories, i.e., difficulties resulting from their poor health condition, and difficulties resulting from hospital conditions and safety protocols (Table 2).

#### 3.1.1. Difficulties Resulting from Poor Health Condition

The following subcategories were identified in this category: 1. Shortness of breath, cough, fever, no appetite; 2. Feeling that you may die; 3. Insomnia and anxiety; 4. Depression; 5. Loss of independence.

Shortness of breath, cough, fever, no appetite

The participants reported symptoms such as shortness of breath, cough, fever, and no appetite.

“For the first few days, I had a terrible cough. I was coughing badly, all the time, all day long. There was not a single moment without coughing. It lasted a few days” (male, age 75, 13 days in hospital).

“I couldn’t eat at all. Tomato juice was the only thing I was able to drink, but not more than 100 mL. And I felt very weak” (female, age 59, six days in hospital).

The participants reported difficulty breathing and weakness as the hardest parts of their stay in hospital.

“The first night in hospital was the hardest. I was weak and had a fever, so I had no appetite. My oxygen saturation was low. A nurse came, opened the window, and helped me get up. I couldn’t get out of bed on my own” (male, age 37, seven days in hospital).

“It was most difficult to breathe. When you don’t have enough oxygen, you begin to suffocate. I was given oxygen therapy. Later, as I went to the bathroom, had a shower and got back, I didn’t have anything... like no power to do anything else. Low oxygen level... it’s hard to describe how you feel” (male, age 70, seven days in hospital).

Feeling that you may die

The health status of some patients was so poor that they were afraid they could die and were in constant fear.

“I was very weak. I was afraid that if I fell asleep, I may never wake up” (female, age 69, 49 days in hospital).

The utterances of some participants were even more dramatic: sometimes they described their experiences as “unimaginable”, or “on the edge”.

“I thought I would not survive. I said goodbye to my family. My wife called, my children called. It’s unimaginable! I could barely endure this” (male, age 62, 10 days in hospital)

“When I heard about sepsis, I thought that was the end. I believed I wouldn’t survive that. Then I lost consciousness and I don’t know what happened next. When I woke up, I couldn’t talk, I could only use gestures. I was afraid I would never talk again, but it was because of the intubation” (female, age 57, 30 days in hospital).

Insomnia and anxiety

Some patients mentioned difficulty sleeping while in hospital though they had never had such problems before. It was often connected with nervousness and anxiety.

“The worst thing was that I couldn’t sleep. I asked for sleeping pills. But if I took the pill in the evening, I was already awake at midnight. So I tried to take them as late as possible, to be able to sleep, but it was a big problem. Apart from that, in the evening I was overstimulated, nervous, and anxious” (male, age 75, 7 days in hospital).

“I couldn’t sleep in hospital. I don’t know, maybe it was stress or something, but I would wake up a few times each night. As I got back home, I slept perfectly the first night, everything was OK, it was home...” (male, age 39, 10 days in hospital).

Depression

The condition of other patients lying in the same or a nearby hospital room affected some participants’ emotions. The participants experienced depression and fear because of the suffering and even deaths of other patients.

“I could hear the voices of patients from other rooms; it was so depressing. It was really unpleasant to listen to those people suffering” (male, age 60, nine days in hospital).

“You know how another person can affect you... I already had pessimistic thoughts because there was a patient next to me, I talked to him. He had the saturation of 90 while I had 70. He was happy that he was already getting better. I talked to him, and then, at night, he died. You know what fear this is. Fear that it happens so quickly! There were a few cases that a patient was lying in the room but later had to be ventilated or taken to another room where they treated more serious cases” (male, age 65, 28 days in hospital).

Other participants, even if they felt bad themselves, were not terrified because they did not see any dramatic scenes.

“Well, it was difficult. I felt bad. But I wasn’t terrified because the people in the same room there were no dramatic situations” (male, age 48, 13 days in hospital).

Loss of independence

Activities such as eating and drinking were also sometimes difficult.

“The first two days were the worst, because I couldn’t do anything on my own. Even sit up in bed to eat. I was lying. I put the plate on my belly and tried to eat a few tablespoons” (female, age 55, 14 days in hospital).

For some patients, assistance with physiological needs was discomforting, and lying in bed for a long time caused them bed sores.

“I couldn’t even raise my arms or legs. I was just lying. I had a catheter and diapers. So, there was some discomfort. I even developed bed sores. I was so helpless. I couldn’t even open a bottle of water. I had no power in my hands. I had to ask someone to do just about everything for me” (female, age 69, 49 days in hospital).

#### 3.1.2. Difficulties Resulting from Hospital Conditions and Safety Protocols

The following subcategories were identified in this category: lack of information about one’s health status, problems with identifying staff members, no empathy from the staff, missing the family, and the sense of slow passing of time.

Lack of physical activity

Being isolated for a long time and not being allowed to leave the hospital room made physical activity impossible, even if the person’s health status was not so bad.

“I was lying in a four-bed room. We were not allowed to leave the room. In fact, the only possibility to move was to walk to and fro in the room to stay mobile, or to lie in bed and raise your legs alternately, left one, right one, left one, right one” (male, age 49, 11 days in hospital).

Lack of direct contact with physicians

It was difficult for the patients to talk directly to the physicians. In some cases the only form of communication with the doctor was over the phone. This made them feel anxious and helpless. The lack of direct contact with physicians also resulted in patients feeling a lack of empathy on the part of doctors.

“The most difficult was the lack of doctors’ help. I was brave and asked for a doctor’s visit, and that was the hardest. I know very well that the doctor’s task is to treat patients. But if he says he has to put on protective clothes to enter the room and then make a record that he put it on, it’s something I can’t understand. The greatest problem was the distance treatment, that the doctor was on the other side of a glass wall and asked me over the phone how I was feeling. I was tearful. Why? Out of these difficult situations, the most difficult was the lack of understanding, lack of empathy” (female, age 67, 11 days in hospital).

Difficulty identifying personnel members

The wearing of protective clothes by the medical personnel made it difficult for patients to differentiate between the individuals taking care of them. Sometimes it caused unpleasant situations, but the participants had a lot of understanding for the medical personnel’s difficulty working in protective clothes and equipment.

“All the workers, doctors, nurses, wore the same clothes. I didn’t know how to address them. Is this an orderly or a nurse? For example, I once asked a priest to bring me a urinal. And he says he’s very sorry but he’s a priest. And nurses in those clothes, like astronauts or penguins, as we sometimes said. Yes, there were strange situations. But I’m not surprised and I sympathize with them, because it’s really hard to work in those clothes” (male, age 65, 28 days in hospital).

Loneliness

For some patients, the most difficult situation was that they missed their family, i.e., children or spouse, and the lack of support from their husband or wife.

“The most difficult for me was that my wife was not beside me. We got married recently. And now we had to be apart. This shocked me the most, that I didn’t have with me my beloved partner I could rely on” (male, age 32, five days in hospital).

“Missing my children, I guess that was the most difficult” (female, age 39, 12 days in hospital).

However, not all the participants had the feeling of loneliness because of the isolation. Some did not find it problematic, depending on their individual situation.

“Being out of home is something I’m used to. I’m often away on business trips so I spend most time away from home. It didn’t affect me much. And now you can always be in touch with people over the phone” (male, age 48, 13 days in hospital).

The sense of slow passing of time

Patients whose health status was good but who had to stay in hospital had problems with finding things to do in their free time and were bored.

“Time slowed down a lot. I couldn’t do anything with the time that passed very, very slow” (male, age 64, seven days in hospital).

“Boredom. This was the most difficult. But for the books I had with me, I would have gone crazy” (male, age 73, 14 days in hospital).

### 3.2. Patients’ Expectations

The patients’ expectations included two main themes: professional care, and support from family members (Table 3).

#### 3.2.1. Professional Care

The participants noticed and highlighted the professionalism of the health care workers. They appreciated both the effects of the treatment and the use of humor, which helped them cope with difficult situations.

“I really received professional care in the hospital. Really, a big thank you to the health care staff... I said to my doctor: ‘Thank you for getting me up’. Despite hard work, they were able to joke, to smile. This helped a lot! I was always emotionally strong, but COVID and the hospital stay made me a bit less resilient” (male, age 75, seven days in hospital).

The participants often experienced kindness and didn’t have any negative comments.

“It was nice when a nurse or doctor came in smiling. Of course, you can’t see anything under those protective clothes, but you can sense the atmosphere. It was always nice, they smiled and joked. The physician would always come when I asked. No problem with that. The nurse would also come any time. So, the staff were really nice” (male, age 70, seven days in hospital).

When describing their stay in hospital, the participants expressed their gratitude to the medical personnel for the care they had received.

“The care was good. The nurses were very nice and helpful. They never refused. I’m very grateful to the physicians and nurses. Really, everything was clean, they cared about it. They changed our diapers. So, I can’t say anything wrong. But of course, the worst thing is you can’t do it yourself and have to use the assistance of other people. But the hospital, the care, nurses, everything was very nice” (female, age 69, 49 days in hospital).

“The nurses and doctors connected with COVID really do a great job. They display a lot of sensitivity and affection, although for them it’s just a routine. I really experienced kindness from them and they were very nice. And I still use their assistance. The doctor who treated me in hospital still cares about my health. It was not just doing his job in hospital... I can say it’s a vocation” (female, age 39, 12 days in hospital).

Some participants could see the shortage of nurses. Even if they did not experience it personally, they saw that other patients were affected by it. This made them feel pity for other patients. But they also explained that situation of shortages in nursing staff.

“In my ward the nurses said there were very few of them and there were many patients, and many of them were non-ambulatory and had to be fed. I was ambulatory, so I could get up and drink some water. But in my room there was a patient who needed to be fed by nurses. And they did not come every time he rang the bell. I felt sorry for those patients who could not get up. There are simply not enough nurses” (male, age 32, five days in hospital).

Some participants strongly emphasized the importance of empathy in care for patients from the very first contact, at the same time understanding that health care professionals can feel burnt out.

“I would like to ask the medical staff to treat patients with greater empathy. As a patient, I need to feel that I’m important, that I’m a human. I would really like to ask the people who treat us to remember this. I know they are burnt out. I teach people about such things. But still, I would like them to think about it more” (female, age 67, 11 days in hospital).

“I remember when I was admitted to hospital, the guys I met were very harsh. The doctor explained that after oxygen therapy my mouth would get very dry. I remember I croaked ‘Water, please!’. And he said ‘All in good time’. I thought ‘Oh well...’ Such coarse treatment prevents you from getting emotional about yourself” (female, age 55, 14 days in hospital).

Health problems and the difficult situation the patients found themselves in caused them to expect care from various specialists, not only a physician and a nurse. This was especially true of patients whose health condition was very poor. Some participants pointed to the importance of receiving care from a psychologist and a physiotherapist, believing that talking to a psychologist would help them feel better and that advice from a physiotherapist would improve their respiratory capacity.

“I would really like a psychologist and a physiotherapist at the ward. They are the most important people. When I was lying in hospital, I was very nervous. If a psychologist had come, talked to me and calmed me down, I would have felt better. If a physiotherapist had visited me before I was discharged and told me how to breathe, it would have been easier” (female, age 67, 11 days in hospital).

After the discharge from hospital, the patients expected reliable information on what they should do next. They emphasized that the information should be provided in writing. They are aware that the Internet is not always a reliable source of data about health issues, and they know how to recognize reliable and unreliable content.

“There should be some guidelines or flyers to hand out to patients. Maybe the Internet is the most popular form of spreading information, but not everybody uses the Internet. Moreover, there are also some things on the Internet that I know were not written by an expert and can’t be treated seriously” (male, age 60, nine days in hospital).

#### 3.2.2. Support

An important and expected element of care is support from loved ones and spiritual support.

The participants appreciated it when they could contact their families with the help of nurses as intermediaries.

“Family support is very important in such situations. The nurses informed me that my family members called and wanted to say hello. They also prayed and even a mass was celebrated for my intention. Such support helps a lot” (female, age 57, 30 days in hospital).

For some patients, spiritual support was essential.

“I pray a lot. I pray a lot all the time. And I prayed in the hospital for my health, because even in the room, everyone prayed” (male, age 75, 13 days in hospital).

## 4. Discussion

We used qualitative research methods to gain insight into the experiences and expectations of patients hospitalized for COVID-19. The experiences voiced by our study participants were similar to ones reported by COVID patients interviewed in other countries, which included physical and mental stress and many negative emotions [1,2]. The difficulties they expressed to us centered around their poor health condition as well as hospital conditions and infection safety protocols.

From the patients’ perspective, the important issues raised were the absence of emotional support from family members and medical personnel, as well as the need for empathy. Lack of empathy from hospital staff was mentioned by many participants. On the one hand, they related it as a negative experience in the hospital and suggested that health care professionals should treat patients with greater empathy. On the other hand, they acknowledged the extraordinary and unprecedented demands of the COVID-19 pandemic on health care teams. A recent study found that if nurses are in a situation of effort–reward imbalance, they may be less empathetic [13]. The authors of that study recommended raising the reward level of nurses as a means of increasing the level of empathy toward patients [13]. Nurses’ behaviors are also a consequence of the effects of working overtime, staff shortages, and dissatisfaction with their job, all factors which have been severely stressed during the pandemic. The improvement of the nurses’ working environment may help improve the quality of the care they provide, even in the pandemic [14].

Patients’ expectations during their hospitalization for COVID-19 focused on professional care, which was understood as the effectiveness of treatment but in the context of staff behaviors such as smiling or telling jokes. A sense of humor is described in the medical literature as an element of trust and respect in the physician–patient relationship, and is an expression of humanity between the patient and the physician [15]. In our study, the patients appreciated the doctors’ ability to joke and claimed this helped them get through the disease.

Despite difficulties with identifying staff members because of their protective clothing and equipment, the patients were sensitive to their non-verbal behaviors and were able to see their smile and sense their mood. Other (quantitative) studies showed that hospital staff were identifiable even when wearing personal protective equipment (PPE), and patients were able to communicate easily with staff despite the PPE [16]. Thus, by using various investigative approaches, both narrative studies and surveys, it is possible to obtain a more complete picture of patients’ experiences [17].

The patients often expressed their gratitude and appreciation of their work to the medical personnel. If there are any shortcomings in nursing care, the patients explained this as being due to nursing staff shortages.

The expectations of patients hospitalized for COVID-19 are a challenge for clinical nurses. Nurses providing care to patients during the COVID-19 pandemic expect preparation, protection and support from nursing leaders [18]. To ensure safe and high-quality care, nurses need to have PPE available and be properly educated in safety procedures [19].

The COVID-19 pandemic poses a serious threat to physical and mental health, not only for inpatients, but also for the general public who have had to stay at home. In a Polish study of 471 subjects confined to home during the pandemic, higher scores of depression, insomnia, loneliness and everyday fatigue were observed [20].

A strong point of our study is that the patients were recruited from several medical facilities and stayed in different hospitals. Thus, they had different experiences resulting from different hospital environments. However, it may be important that the patients reported their experiences of hospitalization when they were already back home, having recuperated from the illness. The time that passed from discharge to the interview differed between participants. The patients’ perspectives might be different if the interviews were conducted, for example, at the end of hospitalization, before the discharge.

Statements of the study participants also show that the medical staff tried to provide the best care to patients despite staff shortages. Certainly, a greater number of doctors, nurses and other medical and non-medical personnel (including clergy) would facilitate meeting the expectations of patients hospitalized due to COVID-19. Larger and more visible identifiers for medical personnel would also be desirable. In addition, the health care system must be adapted for the provision of spiritual support [21].

## 5. Conclusions

Patients hospitalized for COVID-19 experienced both positive and negative emotions. They explained the negative experiences in terms of organizational barriers and medical personnel shortages, especially with regard to clinical nurses. Patients’ expectations are realistic and appropriate to the situation they are in. Apart from professional care, they expect reliable information on what to do after their discharge from the hospital.

Better understanding of patients’ experiences may help clinical nurses provide higher quality care. The results of the study may be used as guidelines on how to improve health care quality and policy.

## Figures and Tables

**Table 1:** Participants’ characteristics (n = 20).

Characteristics	n (%)
Sex	
Female	7 (35)
Male	13 (65)
Age	
32–39	5 (25)
40–49	2 (10)
50–59	3 (15)
60–69	6 (30)
70–75	4 (20)
Education	
Technical	2 (10)
Secondary	10 (50)
University	8 (40)
Hospitalization time (in days)	
5–7	6 (30)
8–14	11 (55)
15–21	0 (0)
22–28	1 (5)
29–35	1 (5)
49	1 (5)

**Table 2:** Patients’ expressions of their most difficult experiences during hospitalization for COVID-19.

Main Categories	Subcategories (n)
Difficulties resulting from their poor health condition	Shortness of breath (5), cough (2), fever (2), no appetite (2)
	Feeling that you may die (5)
	Insomnia and anxiety (4)
	Depression (3)
	Loss of independence (3)
Difficulties resulting from hospital conditions and safety protocols	Lack of physical activity (3)
	Lack of direct contact with physicians (1)
	Difficulty identifying personnel members (2)
	Loneliness (3)
	The sense of slow passing of time (2)

**Table 3:** Hospitalized patients’ expectations.

Main Categories	Subcategories (n)
Professional care	Professionalism of the health care workers (9)
	Kindness (3)
	Availability of a doctor and a nurse (4)
	Empathy (1)
	Presence of a psychologist and physiotherapist in the ward (1)
	Reliable information (1)
Support	Possibility of telephone contact with the family (7)
	Spiritual support (3)

## Data Availability

The datasets for this article cannot be made available by the authors given data protection rules.
